# Cancer Mortality Rates in Female Veterans With and Without TBI

**DOI:** 10.1001/jamanetworkopen.2025.0194

**Published:** 2025-03-13

**Authors:** Christin B. DeStefano, Kelly Cheever, Jeffrey T. Howard, Megan Amuan, Ian J. Stewart, Mary Jo Pugh

**Affiliations:** 1 Walter Reed National Military Medical Center, Bethesda, Maryland; 2 Department of Medicine, Uniformed Services University of Health Sciences, Bethesda, Maryland; 3 Department of Kinesiology, University of Texas at San Antonio; 4 Department of Public Health, University of Texas at San Antonio; 5 Informatics, Decision-Enhancement, and Analytic Sciences Center of Innovation, Veterans Affairs Salt Lake City Health Care System, Salt Lake City, Utah; 6 Division of Epidemiology, Department of Internal Medicine, University of Utah School of Medicine, Salt Lake City; 7 Military Cardiovascular Outcomes Research Program, Uniformed Services University of Health Sciences, Bethesda, Maryland

## Abstract

This cohort study compares cancer mortality rates of female veterans with and without traumatic brain injury (TBI) who served after September 11, 2001, with rates among the general female population of the US.

## Introduction

One in 5 veterans who served after September 11, 2001 (9/11), and served in Iraq or Afghanistan experienced traumatic brain injury (TBI), which is linked to a heightened risk of central nervous system (CNS) cancer and mortality.^1,2^ Despite increasing rates of cancer in female veterans,^3^ data about cancer mortality and the association of prior TBI is lacking. This analysis examined cancer mortality rates between female veterans with and without TBI compared with the US female population.

## Methods

This retrospective cohort study was approved by the University of Utah institutional review board and followed the STROBE reporting guideline. Informed consent was not required because of minimal risk.

This study included 442 715 female military veterans who served on active duty in the US military after 9/11, were aged 18 years or older, and had received 3 or more years of care in the Military Health System (MHS).^1^ At least 2 years of care was required for veterans using the Veteran’s Health Administration.^1^ The cohort was matched to the National Death Index (NDI) for mortality data from 2002 to 2020. Mortality data from the US Centers for Disease Control and Prevention (CDC) WONDER database for 2002 to 2020 were retrieved for the US adult female population.

Veteran and US population cohorts were grouped by age (18-24 years, 25-34 years, 35-44 years, 45-54 years, 55-64 years, and ≥65 years) with aggregate population and death counts. TBI exposure was identified as a positive screening on the Comprehensive TBI Evaluation protocol or medical diagnosis of mild, moderate, severe, or penetrating TBI.^3^ Cancer as underlying cause of death was determined from *International Statistical Classification of Diseases and Related Health Problems, Tenth Revision* codes C00-D48 and grouped as all cancer (C00-D48), breast cancer (C50), CNS cancer (C69-C72), and other cancer (not C50 or C69-C72).

Age-specific cancer mortality rates were estimated with multivariable negative binomial regression models and reported as mortality rates with 95% CIs. Age-adjusted mortality rate ratios (MRRs) with 95% CIs were reported. Statistical significance was set at *P* < .05, and all tests were 2-sided. Analyses were conducted from July 23 to October 1, 2024. Data were analyzed using R version 4.3.2 (R Foundation for Statistical Computing).

## Results

**Figure 1.**
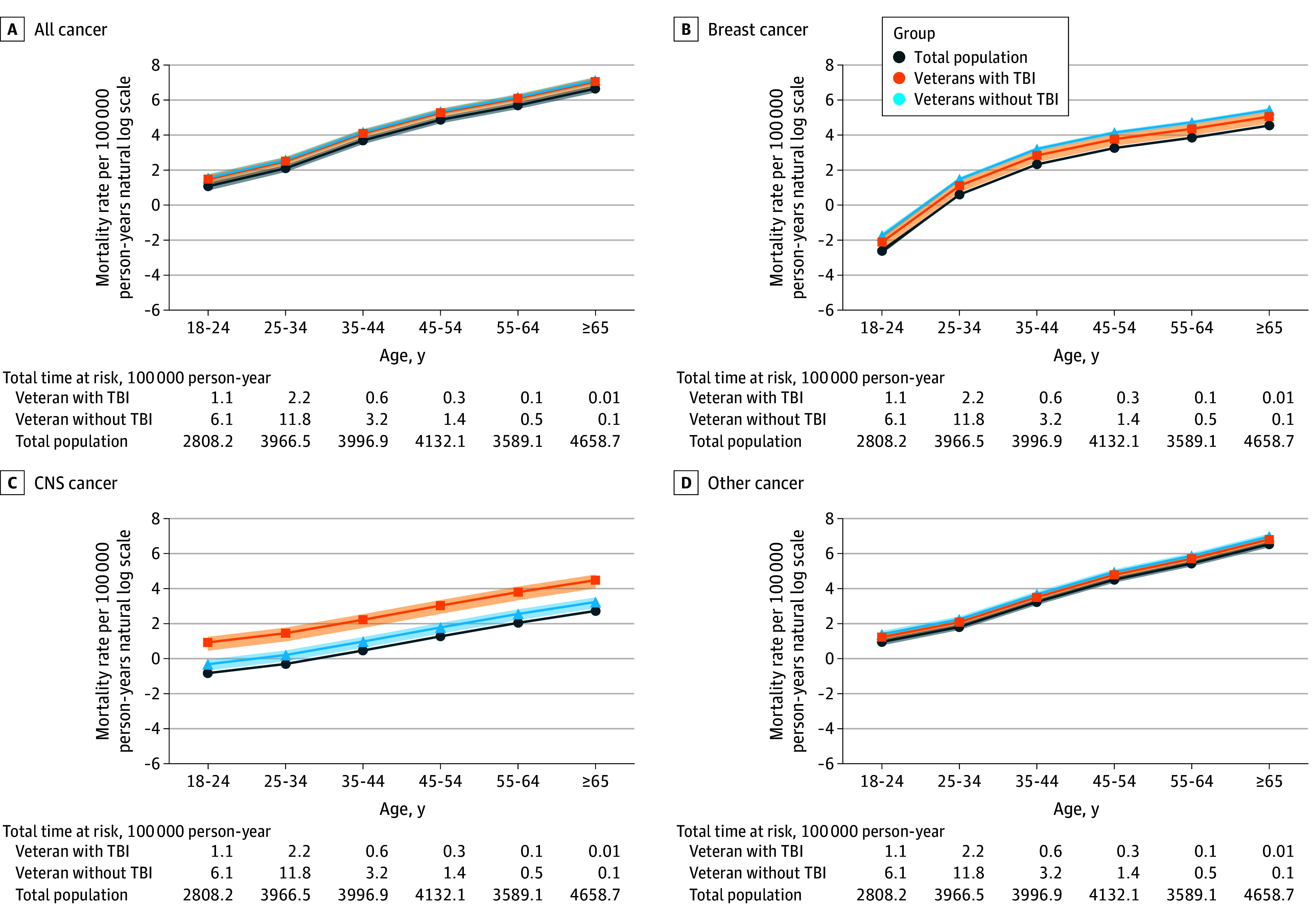
Age-Specific Cancer Mortality Rates Per 100 000 Person-Years for the Total US Adult Female Population and Female Veterans With and Without Traumatic Brain Injury (TBI) by Cancer Type (Natural Log Scale) Compared with the general population, female veterans demonstrated higher cancer mortality rates across all age groups for all cancer types, regardless of TBI. Female veterans with TBI had a particularly increased risk of mortality from central nervous system (CNS) cancer.

The female veteran cohort consisted of 2 729 349 person-years of observation with 1229 cancer deaths, with median (IQR) age upon entry into the cohort of 29 (24-38) years and median (IQR) follow-up time of 10 (7-13) years. There were 5 176 068 deaths among the US female population with 1 237 983 145 person-years of observation. Female veterans showed higher cancer mortality rates across all age groups for all cancer types (Figure 1).

**Figure 2.**
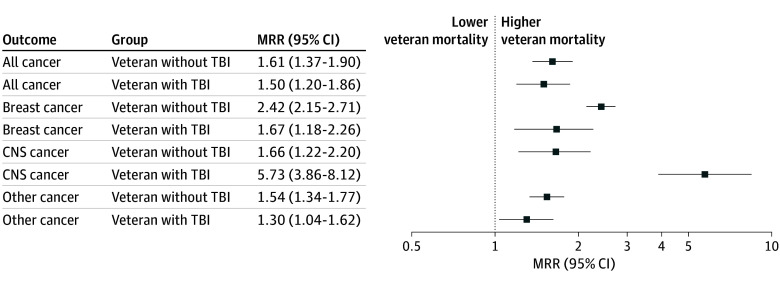
Age-Adjusted Mortality Rate Ratios (MRR), by Type of Cancer for Female Veterans With and Without Traumatic Brain Injury (TBI) Exposure Age-adjusted mortality rate ratios (MRR) show consistently higher cancer mortality for female veterans without TBI for all cancers compared with the total US adult female population.

Female veterans had consistently higher cancer mortality compared with the US female population (Figure 2). Female veterans with and without TBI had similar MRRs for all cancer. However, female veterans without TBI had higher breast cancer mortality (MRR, 2.42; 95% CI, 2.15-2.71) compared with the US female population than female veterans with TBI (MRR, 1.67; 95% CI, 1.18-2.26). Female veterans with TBI had higher CNS cancer mortality (MRR, 5.73; 95% CI, 3.86-8.12) compared with the US female population than female veterans without TBI (MRR, 1.66; 95% CI, 1.22-2.20).

## Discussion

Female veterans who served after 9/11 had greater cancer mortality across all cancer types than age-matched females from the US population. Additionally, female veterans with TBI had a 5.7-fold higher mortality from CNS cancer. Although TBI was not associated with increased breast cancer mortality, female veterans with and without TBI had inferior survival compared with US females. This study is the first to unveil a disparity in cancer mortality in female veterans who served after 9/11 compared with the general population, representing a burden on female veterans and unmet research need. Contributing factors to our findings could include risks associated with military service, cancer stage at diagnosis,^4^ tumor biology, and access to care.^5^ Limitations include potential underestimation of TBI burden and overlap of veteran deaths between the NDI and CDC WONDER databases, which may underestimate effect size.

## Notes

Supplement 1.
eMethods.

eReferences.

Supplement 2.
Data Sharing Statement
