# Error in Key of Figure 1

**DOI:** 10.1001/jamanetworkopen.2025.11599

**Published:** 2025-04-15

In the Research Letter titled “Cancer Mortality Rates in Female Veterans With and Without TBI,”^1^ published March 13, 2025, the colors of the veterans with TBI and veterans without TBI were switched in the key of Figure 1. This article has been corrected.^1^
