# Supplementary material for: Cancer Mortality Rates in Female Veterans With and Without TBI
Source: JAMA Netw Open. 2025 Mar 13;8(3):e250194. doi: 10.1001/jamanetworkopen.2025.0194 (PMC11907312; doi:10.1001/jamanetworkopen.2025.0194)
Supplement: Supplement 1. — eMethods. eReferences. [file jamanetwopen-e250194-s001.pdf]

Supplemental Online Content

DeStefano CB, Cheever K, Howard JT, Amuan M, Stewart IJ, Pugh MJ. Cancer mortality rates in female veterans with and without TBI compared to the US adult females. *JAMA Netw Open*. 2025;8(3):e250194. doi:10.1001/jamanetworkopen.2025.0194

eMethods.
eReferences.

This supplemental material has been provided by the authors to give readers additional information about their work.

© 2025 DeStefano CB et al. *JAMA Netw Open*.

eMethods

Design and Study Setting

This study is a subgroup analysis of females who are part of a retrospectively determined cohort study of mortality rates for military veterans who served active duty in the US military after 9/11 and received care (outpatient, inpatient or prescription) in the Department of Defense (DoD) Military Health System (MHS) with/without care in the Veteran's Health Administration (VHA). This study is part of the Long-Term Impact of Military-Relevant Brain Injury Consortium and Chronic Effects of Neurotrauma Consortium (LIMBIC-CENC) Phenotype Study, which aims to examine health trajectories of veterans with and without traumatic brain injury (TBI) longitudinally for individuals with care in DoD only and with both DoD and VA care. To accomplish this, veterans were required to have 3+ years of DOD care, and 2+ years of VHA care for those that entered the VHA healthcare system. Since military operations following 9/11 began in October 2001, no military veterans met criteria for the study until 2002. However, all individuals eligible in 2002 had to have 3 years of care in the MHS, meaning that the MHS care eligibility requirement was examined beginning in 1999. The criterion for 3 or more years of care in the MHS was not required to be 3 or more years of care following a TBI. Individuals were eligible for inclusion upon their third year of care in the MHS, if they were alive and 18 years of age or older. For veterans with TBI, the cohort entry index date used for calculating follow-up time is the date of TBI diagnosis, and for veterans without TBI, index dates were simulated by Monte Carlo methods to model age-correlated dates of first diagnosis in the TBI cohort and using the model to simulate index dates for age-matched controls. Total follow-up time is the difference between the index date (exposure) and the date of death from the NDI or

12/31/2020 for censored observations. The derivation of the full study cohort is described in a previous publication.[1]

The data for the female subgroup from this cohort were used for this study. The goal with this analysis was to quantify cancer mortality rates for female military veterans with and without TBI exposure, and to compare these two groups to the overall cancer mortality rates for the total US adult female population. Based on prior findings,[1] we hypothesized that female veterans would have higher cancer mortality rates than the total US adult female population and that female veterans with TBI exposure would have higher cancer mortality than female veterans without TBI. The research protocol was approved by the institutional review board and conducted in accordance with applicable Federal regulations. This study followed the Strengthening the Reporting of Observational Studies in Epidemiology (STROBE) reporting guideline for cohort studies.

Data Sources

Demographic and deployment data for veterans were retrieved from the Veterans Affairs/Department of Defense Identity Repository (VADIR) database, along with matching health records data from the MHS Management Analysis and Reporting tool (M2) and the VHA Veterans Informatics and Computing Infrastructure (VINCI) and combined with data from the National Death Index (NDI) for mortality follow-up through December 31, 2020. The individual level data were aggregated into a period-specific dataset based on the year of entry into the cohort and the duration of follow-up time, calculated as the difference between the cohort entry date and the date of death or censoring. For example, an individual who entered the cohort in 2010 and died in 2020 would appear in the population counts for each year from 2010 through 2020 and would appear in the death count for year 2020 in the resulting aggregate dataset.

© 2025 DeStefano CB et al. *JAMA Netw Open*.

Population and death counts for the total US adult female population were compiled from 2002-2020 from the Centers for Disease Control and Prevention (CDC) WONDER database.[2] Population and death counts were aggregated by each value of year, age group and TBI exposure. This created 3 groups for comparison, female veterans with TBI exposure, female veterans without TBI exposure and the total US adult female population.

Measures

Demographic variables for age, sex and race/ethnicity were included in this study. Age was measured as 10-year age groups (18-24, 25-34, 35-44, 45-54, 55-64, 65+ years of age). Each year was coded as numeric year, from 2002-2020.

Military veterans were categorized as no TBI if there was no known record of either a positive TBI screening from the Comprehensive TBI Evaluation (CTBIE) protocol, or a medical record of diagnosis of mild, moderate, severe or penetrating TBI. Severity of TBI was not assessed in this study. For military veterans without a positive CTBIE screening, International Classification of Disease, versions 9 and 10 (ICD) Clinical Modification (CM) codes were used to identify TBI exposure consistent with the Armed Forces Health Surveillance Branch (AFHSB) definitions. A full list of all ICD9 and ICD10 CM codes used for TBI ascertainment can be found in a previously published article in JAMA Network Open.[1]

Statistical Analysis

Age-specific mortality rates per 100,000 person-years and age-adjusted mortality rate ratios (MRR) per 100,000 person years were estimated using negative binomial regression models for both the military veteran cohort and the total US adult female population. Covariates included age groups, year (period) and comparison group (veteran with TBI, veteran without TBI

and total US population). Mortality rates, MRR and 95% confidence intervals (CI) are reported graphically. Data were analyzed using R version 4.3.2 (R Foundation for Statistical Computing).

eReferences

1. Howard JT, Stewart IJ, Amuan M, Janak JC, Pugh MJ. Association of traumatic brain injury with mortality among military veterans serving after September 11, 2001. JAMA Netw Open. 2022;5(2):e2148150. doi:10.1001/jamanetworkopen.2021.48150.
2. National Center for Health Statistics Division of Vital Statistics. CDC WONDER: Underlying Cause of Death, 2002-2020. 2022. Accessed July 23, 2024. https://wonder.cdc.gov/ucd-icd10.html.

© 2025 DeStefano CB et al. *JAMA Netw Open*.
